# Polymorphisms of the kappa casein (*CSN*3) gene and inference of its variants in water buffalo (*Bubalus bubalis*)

**DOI:** 10.5194/aab-62-585-2019

**Published:** 2019-12-05

**Authors:** Xinyang Fan, Zifang Zhang, Lihua Qiu, Yongyun Zhang, Yongwang Miao

**Affiliations:** 1 Faculty of Animal Science and Technology, Yunnan Agricultural University, Kunming 650201, Yunnan, China; 2 Department of Animal Husbandry and Veterinary medicine, Yunnan Agricultural College of Vocational Education, Kunming 650212, Yunnan, China; 3 Teaching Demonstration Center of the Basic Experiments of Agricultural Majors, Yunnan Agricultural University, Kunming 650201, Yunnan, China

## Abstract

Kappa casein plays a crucial role in the formation of stable casein micelles and has a key influence on milk-clotting properties. However, current understanding of buffalo *CSN*3 gene polymorphisms is not sufficient. In this study, the polymorphisms in the complete coding sequence (CDS) of the buffalo *CSN*3 were detected using PCR product direct sequencing. The CDS of *CSN3* for river and swamp buffalo was the same in length, which contained an open reading frame of 573 nucleotides encoding a peptide containing 190 amino acid residues. A total of eight single nucleotide polymorphisms (SNPs) was identified in two types of buffalo. Among them, c.86C>T, c.252G>C, c.445G>A, c.467C>T and c.516A>C were non-synonymous, which leads to p.Pro8Leu, p.Lys63Asn, p.Val128Ile, p.Thr135Ile and p.Glu151Asp substitutions in buffalo kappa casein (κ-CN), respectively. The substitution of p.Thr135Ile may exert a vital effect on the function of buffalo κ-CN. Eleven haplotypes were defined based on the SNPs found in buffalo, and accordingly, seven protein variants and four synonymous variants of buffalo κ-CN were inferred, called variants A, B, B${}^{1}$, C, C${}^{1}$, C${}^{2}$, D, E, F, F${}^{1}$ and G. The variants observed in water buffalo did not exist in the *Bos* genus. In addition, 14 amino acid differential sites of κ-CN between buffalo and the *Bos* genus were identified, of which 3 were located at glycosylation sites (80S, 96T, 141S) and 4 at phosphorylation sites (19S, 80S, 96T, 141S). It is speculated that they may lead to differences in the physicochemical properties of κ-CN between buffalo and the *Bos* genus. This study will lay a foundation for exploring the association between the variation in the *CSN3* gene and the lactation traits of buffalo.

## 1 Introduction

Kappa casein (CSN3, κ-CN), as one of the main components of milk protein in mammals, plays an important role on casein micelle stability (Komori et al., 2013). κ-CN is situated predominantly on the surface of casein micelle in milk. The hydrolytic activity of chymosin can split κ-CN into the insoluble para-κ-CN and the soluble hydrophilic glycopeptide (macro-peptide). As a result, destabilized casein micelles precipitate in the form of a gel (Hobor et al., 2008). This is a crucial process in milk coagulation not only for cheese making but also for the nutrition of suckling calves (Caroli et al., 2009). In addition, κ-CN is the only glycosylated casein for eutherians, which contains the sulfur amino acids cysteine and methionine. κ-CN can be attached to carbohydrates via O-glycosidic linkages to its threonine and serine residues (Ginger and Grigor, 1999; Matějíček et al., 2008). Nutritionally, κ-CN supplies the sucking neonate with amino acids, highly bioavailable calcium and potential bioactive peptides (Caroli et al., 2009).

The *Bos taurus CSN3* gene has been mapped on BTA 6 which contains five exons and four introns. Its complete coding sequence (CDS) is 573 bp in length, encoding a peptide composed of 190 amino acid residues, of which the first 21 amino acid residues form a signal peptide (Ward et al., 1997). The κ-CN variants have been extensively studied in the *Bos* genus for decades. To date, 13 protein variants (A, B, B${}^{2}$, C, D, E, F${}^{1}$, F${}^{2}$, G${}^{1}$, G${}^{2}$, H, I and J) and 1 synonymous variant (A${}^{1})$ of κ-CN in the *Bos* genus have been identified at the protein and DNA levels (Farrell et al., 2004; Chen et al., 2008; Caroli et al., 2009). Previous studies have primarily focused on its two most common genetic variants (A and B), which have been shown to be related to differences in milk composition or yield and curdling characteristics of rennet in animals of *Bos taurus* (Lara et al., 2002; Tsiaras et al., 2005; Alipanah et al., 2008).

Water buffalo (*Bubalus bubalis*) are of great economic significance in tropical and subtropical countries for their dairy, meat and draught purposes (Michelizzi et al., 2010). Domestic buffaloes are divided into river and swamp buffalo. The former is mainly used for milk production, which produces about 2000 kg milk per year, while the latter is mainly used for draught and produces 500–600 kg milk per year. At present, more than 5 % of the milk produced around the world is supplied by water buffalo (Michelizzi et al., 2010). Moreover, the proportion of buffalo milk has increased over the years, suggesting a preference for water buffalo as milking animals (Dayem et al., 2008). κ-CN accounts for approximately 12 % of the total casein in buffalo milk. It represents one component of major milk proteins which is involved in the formation of casein micelles and determines the size and function of micelles (Mukesh et al., 2006; Ahmad et al., 2013). To date, the buffalo *CSN3* gene has been mapped on BBU 7 (Lannuzzi et al., 2003), and its untranslated regions (UTRs) and coding region sequences are available (AM900443) (Masina et al., 2007). In contrast to cattle, the polymorphisms of the *CSN3* gene in water buffalo are less extensively investigated. Most of the existing studies in buffalo were limited to the genotyping of variants A and B found in dairy cows using PCR restriction fragment length polymorphism, PCR single-strand conformation polymorphism and DNA sequencing (Othman, 2005; Gangaraj et al., 2008; Nahas et al., 2013). Although four genetic variants of κ-CN have been identified in river buffalo (two at the exon II and two at the exon IV), complete information about the variants of κ-CN in buffalo is still rare (Mitra et al., 1998; Bonfatti et al., 2012); in particular, the polymorphisms of the *CSN3* gene in swamp buffalo have not been reported yet. The objective of this study is to identify and characterize the polymorphisms of the *CSN3* gene in river buffalo and swamp buffalo at the DNA level, further to identify new κ-CN variants in the two types of buffalo, and to establish the phylogenetic relationship of κ-CN variants between buffalo and the species of the *Bos* genus.

## 2 Materials and methods

### 2.1 DNA sample collection

A total of 384 blood samples were assayed in this study, including 174 river buffaloes (24 Murrah, 51 Nili-Ravi and 99 Binglangjiang buffalo) and 210 swamp buffaloes (21 Fuzhong, 33 Xilin, 24 Dehong, 33 Diandongnan, 27 Yanjin, 30 Fu'an, 24 Dongliu and 18 Guizhou buffalo). All samples were collected at random, and there was no direct kinship between individuals. The samples were transported by cryopreservation and then stored in a refrigerator at -80 ${}^{\circ }$C in the laboratory.

Murrah and Nili-Ravi are two buffalo breeds introduced into China. Samples of these two types of buffalo were collected from the livestock frozen semen station in Yunnan province, China. Binglangjiang buffalo is the first indigenous river buffalo breed found in Yunnan province in China in recent years. The buffalo samples were collected from the Binglangjiang buffalo breeding base in Tengchong City, Yunnan Province, China. Samples of various swamp buffaloes were collected from the main distribution areas of China, where they are located.

In accordance with the Guide for Animal Care and Use of Experimental Animals, all procedures for sample collection were performed and approved by the Institutional Animal Care and Use Committee of Yunnan Agricultural University.

Genomic DNA was extracted from the samples according to a standard proteinase-K and phenol–chloroform method (Sambrock and Russell, 2001), and the concentration and purity were detected using gel electrophoresis and a NanoDrop 2000 UV-Vis spectrophotometer (Thermo Fisher Scientific, Waltham, MA, USA). Then, the genomic DNA was diluted to 50 ng µL${}^{-1}$ using TE buffer and stored at 4 ${}^{\circ }$C.

### 2.2 PCR and sequencing

The exons and their flanking sequences of the buffalo *CSN3* gene were amplified with the primers listed in Table 1. All primers were designed based on the complete sequence of the buffalo *CSN*3 (accession no. AM900443).

**Table 1:** Primer information in this study.

Primer name	Distribution	Primer	Product	Annealing
	region	sequences	length (bp)	temperature (${}^{\circ }$C)
*CSN3*-F1-U	915–932	CCGAGACTGATGTAAAGA	346	50
*CSN3*-F1-L	1241–1260	CATAATGATGACAAAGGATA		
*CSN3*-F2-U	3330–3349	GATCAACCTTGTAATGACTC	484	51
*CSN3*-F2-L	3794–3813	CTTCTTTATTCCTAGAAACC		
*CSN3*-F3-U	9361–9380	TAACCTAGAAAAGTGCTTTA	445	50
*CSN3*-F3-L	9786–9805	CTCATGTTGCTAAATACTCA		
*CSN3*-F4-U	11669–11692	CGCTGTGAGAAAGAGGAAAGATTC	779	59
*CSN3*-F4-L	12422–12447	AGATTCAAGGAGTATACCAATTGTTG		
*CSN3*-F5-U	13954–13973	ATGAAGCAAATCACGGAAGC	427	56
*CSN3*-F5-L	14361–14380	TGACCCAGAGGGATGGTATG		

PCR was performed in a final 25 µL reaction mixture containing 2.0 µL of DNA template, 2.5 µL of 10 × Taq DNA polymerase buffer (${\mathrm{Mg}}^{2+}$ Plus), 0.4 µL of 10 mM of each primer, 2.0 µL of 25 mM dNTPs (deoxy-ribonucleoside triphosphates) (TaKaRa, Dalian, China), 0.3 µL of 5 U µL Taq DNA polymerase (TaKaRa, Dalian, China) and 17.4 µL of sterile water. The mixture was denatured for 5 min at 95 ${}^{\circ }$C, and PCR was run for 34 cycles at 95 ${}^{\circ }$C for 40 s (denaturation), 50–59 ${}^{\circ }$C for 40 s (annealing), 72 ${}^{\circ }$C for 45 s (extension) and a final extension for 5 s min at 72 ${}^{\circ }$C. PCR products were detected using 2 % agarose gel electrophoresis stained with ethidium bromide. Then, the PCR products were sequenced in both directions using Sanger sequencing.

### 2.3 Data analysis

The sequences of buffalo *CSN*3 obtained in this study were checked, edited and assembled by the Lasergene software package (DNAstar, Inc.). The open reading frame (ORF) was determined exploiting the program ORF Finder (http://www.ncbi.nlm.nih.gov/gorf/, last access: 6 July 2019) and translated into an amino acid sequence. Physicochemical characteristics including isoelectric point and theoretical molecular weight, hydropathy, the O-glycosylation site, the phosphorylation site and signal peptide were predicted by the ComputepI/Mw tool (http://web.expasy.org/compute_pi/, last access: 6 July 2019; Walker, 2005), the ProtParam tool (http://web.expasy.org/protparam/, last access: 6 July 2019), the NetOGlyc 4.0 Server (http://www.cbs.dtu.dk/services/NetOGlyc/, last access: 6 July 2019), the NetPhos 3.1 Server (http://www.cbs.dtu.dk/services/NetPhos/, last access: 6 July 2019) and the SignalP software (http://www.cbs.dtu.dk/services/SignalP/, last access: 6 July 2019; Petersen et al., 2011), respectively. The single nucleotide polymorphisms (SNPs) were checked and outputted by Seqman (DNAstar, Inc.) and Mega 7 (Kumar et al., 2016) softwares. The estimate of genotype frequencies and allele frequencies and the Hardy–Weinberg balance test were conducted for each SNP using the PopGene 1.32 software (Yeh and Boyle, 1997). Based on the Bayesian model algorithm, the haplotypes with high confidence were deduced by the PHASE software, and the number of iterations is ≥100 (Stephens et al., 2001). The effect of amino acid substitutions on the protein function was evaluated by PROVEAN (http://provean.jcvi.org/index.php, last access: 6 July 2019). The default threshold for this program is -2.5. Amino acid substitutions with a score equal to or below -2.5 are considered deleterious to the protein, and amino acid substitutions with a score above -2.5 are considered neutral (Choi and Chan, 2015).

To investigate the probable phylogenetic relationship between the haplotypes of buffalo *CSN3*, median-joining networks were constructed using the program Network 4 (http://www.fluxus-engineering.com, last access 7 July 2019). Furthermore, the phylogenetic trees based on the haplotype sequences of the *CSN3* gene were constructed using the Mega 7 software. The maximum-likelihood (ML) tree was constructed by adopting the Hasegawa–Kishino–Yano model after model selection tests. The robustness of the nodes was evaluated by the bootstrap method after 10 000 replications.

## 3 Results

### 3.1 PCR and sequence analysis

**Figure 1:**
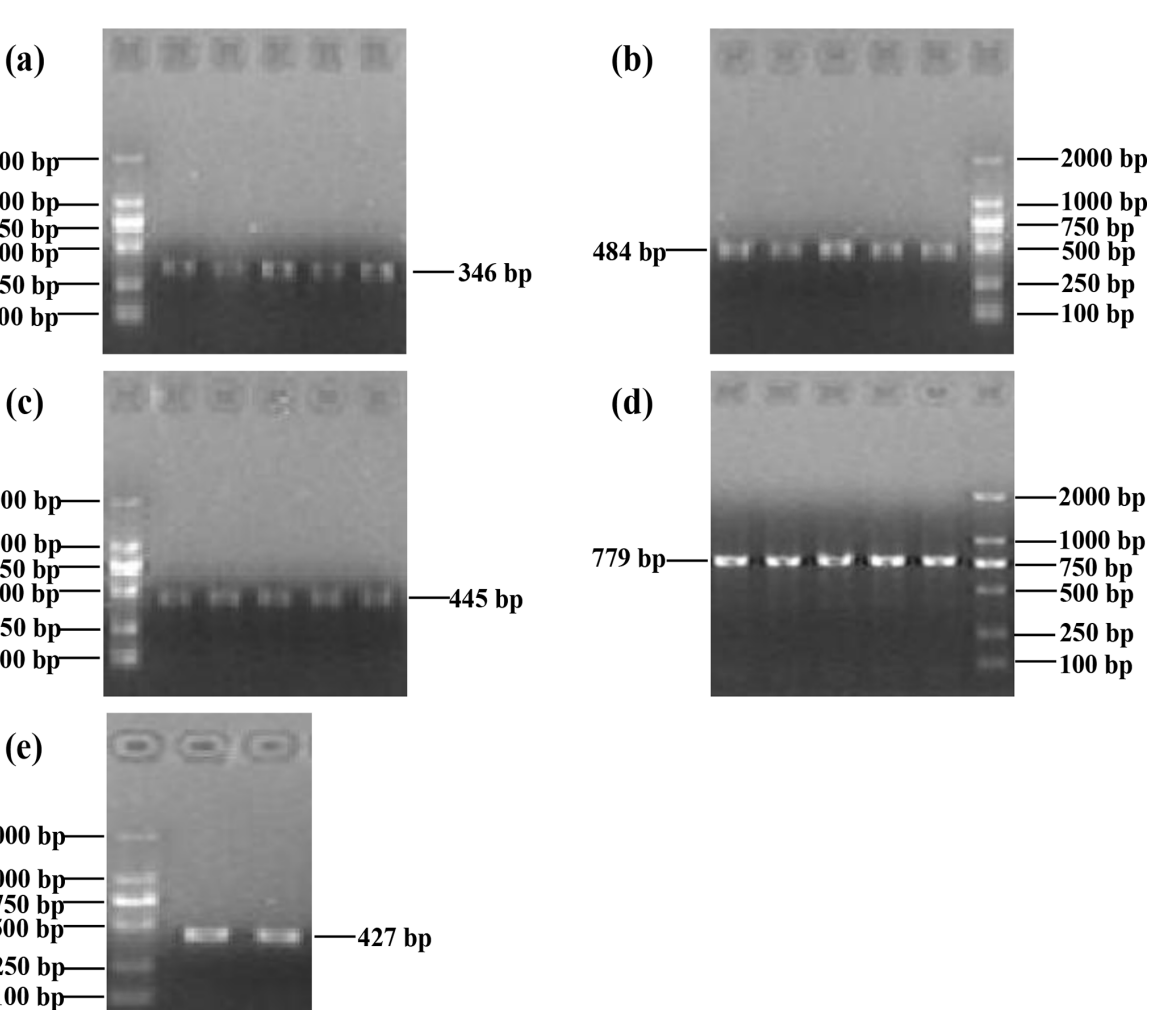
Five PCR-amplified fragments of buffalo *CSN3* detected by agarose gel electrophoresis, covering exon I **(a)**, exon II **(b)**, exon III **(c)**, exon IV **(d)** and exon V **(e)** in turn.

The PCR products covered the five exons of buffalo *CSN3*, i.e., 346, 484, 445, 779 and 427 bp. The results of agarose gel electrophoresis are shown in Fig. 1.

**Figure 2:**
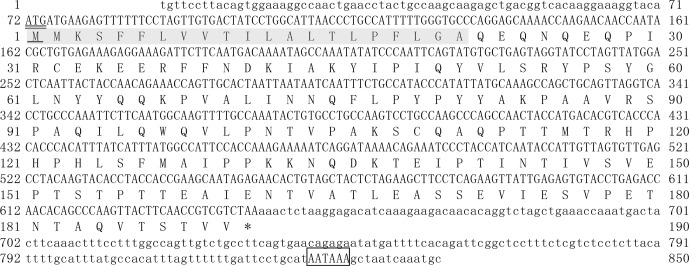
Nucleotide sequence of buffalo *CSN*3 and deduced amino acid sequence; $5^{\prime }$ and $3^{\prime }$-untranslated regions are written in lowercase and ORF is in uppercase. The predicted protein sequence is shown immediately below the nucleotide sequence. The start codon is double underlined and its amino acid is underlined. The signal sequence is shaded. The stop codon is indicated by an asterisk (${}^{*}$). The polyadenylation signal is boxed.

PCR products of five fragments were subjected to bi-directional sequencing. The assembled sequence was compared with the bovine homologous sequence published in the NCBI database (https://www.ncbi.nlm.nih.gov/, last access: 6 July 2019) to confirm their correctness. The obtained sequences were 2348 nucleotides in length, including 848 nucleotides of exon sequences and 1500 nucleotides of exon flanking sequences. The results showed that the CDS length and the structure of the buffalo *CSN*3 gene were the same as those of the species in the *Bos* genus. The exon lengths of this gene from exon I to exon V were 64, 62, 33, 517 and 172 bp, respectively. The $5^{\prime }$-UTR consisted of 69 nucleotides, and 3'-UTR was composed of 206 nucleotides. The full length of buffalo *CSN3* CDS is 573 nucleotides, i.e., 57 nucleotides of exon II, 33 nucleotides of intact exon III and 483 nucleotides of exon IV. The mean nucleotide composition of buffalo *CSN3* CDS consists of 33.16 % A, 16.58 % G, 24.61 % T and 25.65 % C. Buffalo *CSN3* was predicted to encode a protein composed of 190 amino acids (AAs) with a 21-AA signal peptide at N terminus and a mature peptide of 169 AAs (Fig. 2). In the mature peptide, the AA 1–105 at N terminus is hydrophobic, while the AA 106–169 at C terminus is hydrophilic and will be released as a macro-peptide into the whey after chymosin hydrolysis of milk κ-CN during cheese production. The molecular weight of the mature peptide for buffalo κ-CN is about 19.10 kDa and the theoretical isoelectric point is 6.35. The grand average of hydropathicity (GRAVY) was -0.526, which suggests that the mature buffalo κ-CN is a hydrophilic protein. The predicted results revealed that 13 O-glycosylation sites and 19 phosphorylation sites existed in the mature peptide of buffalo κ-CN, which were different from those of cattle κ-CN in number and location (Table 2).

**Table 2:** Modification sites of the κ-CN mature peptide in buffalo and cattle.

Modification	Buffalo${}^{*}$	Cattle
O glycosylation	*82T*, 87S, 93T, 94T, *96T*, *121T*, 127S, 131T, 132S, 133T, 135T, 136T, 142T	*80S*, 87S, 93T, 94T, 112K, *124T*, 127S, 131T, 132S, 133T, 135T, 136T, *141S*, 142T, *145T*
Phosphorylation	37S, 61Y, 69S, 82T, 87S, *96T*, 104S, *124T,* 127S, 131T, 132S, 133T, 136T, 145T, 149S, *150S*, 155S, 161T, *167T*	*19S*, 37S, 61Y, 69S, *80S*, 82T, 87S, *93T*, *94T*, 104S, 127S, 131T, 132S, 133T, 136T, *141S*, *142T*, 145T, 149S, 155S, 161T

${}^{*}$ The specific modification sites of species are italicized.

### 3.2 Population genetic analysis

In the samples of this study, a total of five SNPs were identified in the two types of buffalo, in which the substitution c.86C>T was located in exon III and c.445G>A, c.467C>T, c.471C>T (>G) and c.516A>C in exon IV. Four substitutions were all shared by river and swamp buffalo except that the c.467C>T was detected only in river buffalo (all the swamp buffalo were homozygous CC; the type c.467T was not found in the swamp buffalo). Among the five SNPs, c.86C>T, c.445G>A, c.467C>T and c.516A>C were non-synonymous, leading to changes in their corresponding amino acid in the mature peptide: p.Pro8Leu, p.Val128Ile, p.Thr135Ile and p.Glu151Asp. The p.Pro8Leu, p.Val128Ile and p.Glu151Asp belonged to similar amino acid substitutions in physicochemical properties, while p.Thr135Ile belonged to the substitution with different physicochemical properties. SNP471 was located at the third nucleotide of codon 136 of the mature peptide and had two synonymous substitution (c.471C>T and c.471C>G). Among them, c.471G appeared at a very low frequency (0.0172) and existed only in heterozygotes TG and CG.

The genotype frequency, allele frequency and Hardy–Weinberg balance test for each SNP locus are presented in Table 3. The c.86C, c.445G and c.471C were high-frequency alleles at corresponding loci in two types of buffalo. In river buffalo, the c.467C allele had the highest frequency (0.9224), while SNP467 was homozygous in swamp buffalo with homozygote of the CC type. It is noteworthy that allele frequencies of SNP516 were significantly different between river and swamp buffalo. The Hardy–Weinberg balance test showed that SNP445, SNP467 and SNP471 in river buffalo and SNP516 in swamp buffalo were in an unbalanced state (P<0.05).

**Table 3:** Polymorphic loci and allelic and genotypic frequencies in two types of buffalo.

Population	SNP	Genotype frequency		Allele frequency		P value${}^{*}$
		Genotype	Frequency	Allele	Frequency	
River buffalo	c.86C>T	CC	0.929	C	0.9643	1.00000
		CT	0.071	T	0.0357	
		TT	0.000			
	c.445G>A	GG	0.948	G	0.9655	0.00002
		GA	0.034	A	0.0345	
		AA	0.017			
	c.467C>T	CC	0.879	C	0.9224	0.00119
		CT	0.086	T	0.0776	
		TT	0.034			
	c.471C>T (>G)	CC	0.862	C	0.9052	0.00098
		CT (CG)	0.069 (0.034)	T (G)	0.0776 (0.0172)	
		TT	0.034			
	c.516A>C	AA	0.914	A	0.9569	0.75994
		AC	0.086	C	0.0431	
		CC	0.000			
Swamp buffalo	c.86C>T	CC	0.625	C	0.8125	0.60385
		CT	0.375	T	0.1875	
		TT	0.000			
	c.445G>A	GG	0.900	G	0.9500	0.68453
		GA	0.100	A	0.0500	
		AA	0.000			
	c.467C>T	CC	1.000	C	1.0000	-
		CT	0.000	T	0.0000	
		TT	0.000			
	c.471C>G	CC	0.971	C	0.9857	0.93192
		CG	0.029	G	0.0143	
		GG	0.0			
	c.516A>C	AA	0.271	A	0.4429	0.00894
		AC	0.343	C	0.5571	
		CC	0.386			

${}^{*}$ P value of Hardy–Weinberg equilibrium test.

**Figure 3:**
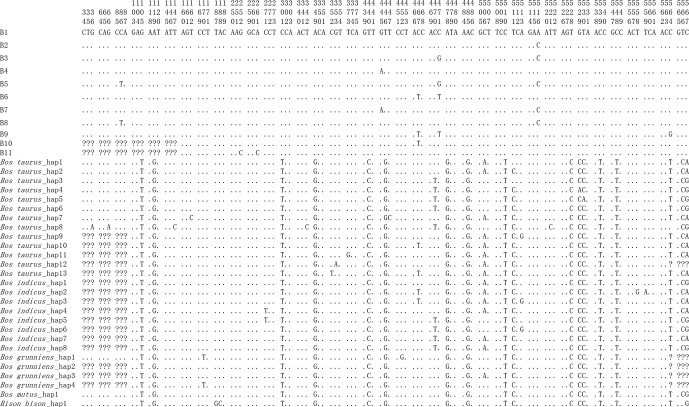
Nucleotide differences in the haplotype sequences between buffalo and the species of the *Bos* genus. Number represents the position of coding region. Dots (⋅) represents identity with B1. Nucleotide substitutions are denoted by different letters. Missing information is indicated by a question mark (?).

Combining the data in this study with buffalo *CSN3* data published in NCBI, it is found that the number of polymorphic loci in buffalo increased to eight, of which four were shared by river and swamp buffalo. The SNP252, SNP261, SNP467 and SNP564 were observed only in river buffalo (Fig. 3).

### 3.3 Haplotypes division and their genetic relationship

**Figure 4:**
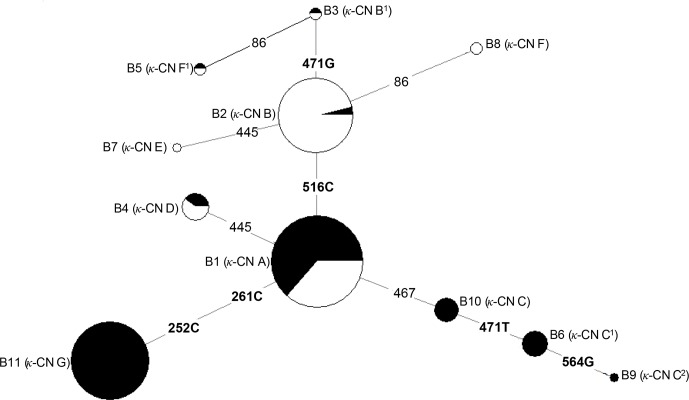
Network profile of 11 buffalo haplotypes of the buffalo *CSN3* gene. B1–B11 are the haplotypes defined in water buffalo. Mutations along the branch are labeled by the nucleotide positions; transversions are specified by further adding suffixes A, G, C and T. Each haplotype is represented by a circle, with the area of the circle proportional to its frequency. Samples from river and swamp buffalo are indicated by black and white color, respectively.

Based on the observed eight SNPs, 11 haplotypes were defined in buffalo (B1–B11) (Figs. 3, 4). Among them, eight haplotypes (accession numbers MF679163-MF679170) were determined by the data in this study, and the other three haplotypes were from online data (accession numbers EH119177, JQ670673, FJ770200) (Mukesh et al., 2006; Riaz et al., 2008; Nahas et al., 2013). Among the eight haplotypes identified in this study, B1–B6 haplotypes actually existed in the detected samples, while B7–B8 were inferred only by the PHASE program. The expected and actual frequencies of these haplotypes are shown in Table 4. Among the 11 haplotypes, B1–B5 were shared by river and swamp buffalo. The B1 was the main haplotype of river buffalo with a frequency of 0.84, but its frequency was only 0.40 in swamp buffalo. The frequency of B2 was 0.52 in swamp buffalo and 0.03 in river buffalo. The haplotypes B6, B9, B10 and B11 were specific to river buffalo, while B7 and B8 were exclusive to swamp buffalo. The alignment by the haplotype sequences showed that there were no nucleotide sites that could distinguish the two types of buffalo.

**Table 4:** Haplotype information of buffalo *CSN3*.

	Haplotype	Actual	Expected
		frequency	frequency
B1	CGCCA	0.613	0.611
B2	CGCCC	0.287	0.284
B3	CGCGC	0.008	0.007
B4	CACCA	0.040	0.034
B5	TGCGC	0.008	0.007
B6	CGTTA	0.032	0.032
B7	CACCC	0.004	0.004
B8	TGCCC	0.008	0.021

The genetic relationship among 11 haplotypes of buffalo *CSN3* were constructed by the median-joining network method (Fig. 4). Eleven buffalo haplotypes were divided into two groups on the median-joining network. One group, including B2, B3, B5, B7 and B8, was mainly distributed among swamp buffalo with haplotype B2 as the central one, and the other group, including haplotypes B1, B4, B6, B9, B10 and B11, was mainly found in river buffalo with B1 as the central haplotype. There are one or two mutations between B2 and B3, B5, B7 and B8. Therefore, it is concluded that B3, B7 and B8 have probably evolved from a single mutation of B2, and B5 may have evolved from two mutations of B2. Except for only one mutation between B1 and B4, B10, there were two or more mutations between B1 and its derived haplotype. Therefore, B4 and B10 may have evolved from single mutation of B1, B6 and B11 may be evolved from B1 through two mutations, and B9 through three mutations.

In terms of haplotype composition and frequency distribution of the *CSN3* gene, there were obvious differences between the two types of buffalo, revealing that the *CSN3* gene in the two types of buffalo had different evolutional patterns. In view of the fact that B1 is widely distributed in two types of buffalo and B2 is mainly distributed in swamp buffalo, it can be inferred that the haplotype B1 may represent the ancestral haplotype of buffalo *CSN3*, while other haplotypes may originate from this haplotype. Whether this is the case, it is necessary to further expand the sample for experimental verification.

### 3.4 Nucleotide differences between buffalo and the species of the *Bos* genus

In order to reveal the difference of CDS of the *CSN3* gene between buffalo and the species of the *Bos* genus, the published homologous sequences of the *Bos* genus were included in this study. A total of 117, 12, 5, 3 and 3 sequences of *Bos taurus*, *Bos indicus*, *Bos grunniens*, *Bos mutus* and *Bison bison* were recruited from the NCBI database, respectively. Accordingly, 13, 8, 4, 1 and 1 haplotypes were defined in these species, respectively (Fig. 3). The alignment of all haplotype sequences displayed 15 nucleotide differences between buffalo and the species of the *Bos* genus, which were found in the sequences at positions c.105, c.119, c.301, c.349, c.440, c.446, c.478, c.485, c.510, c.528, c.529, c.530, c.539, c.548 and c.567.

**Figure 5:**
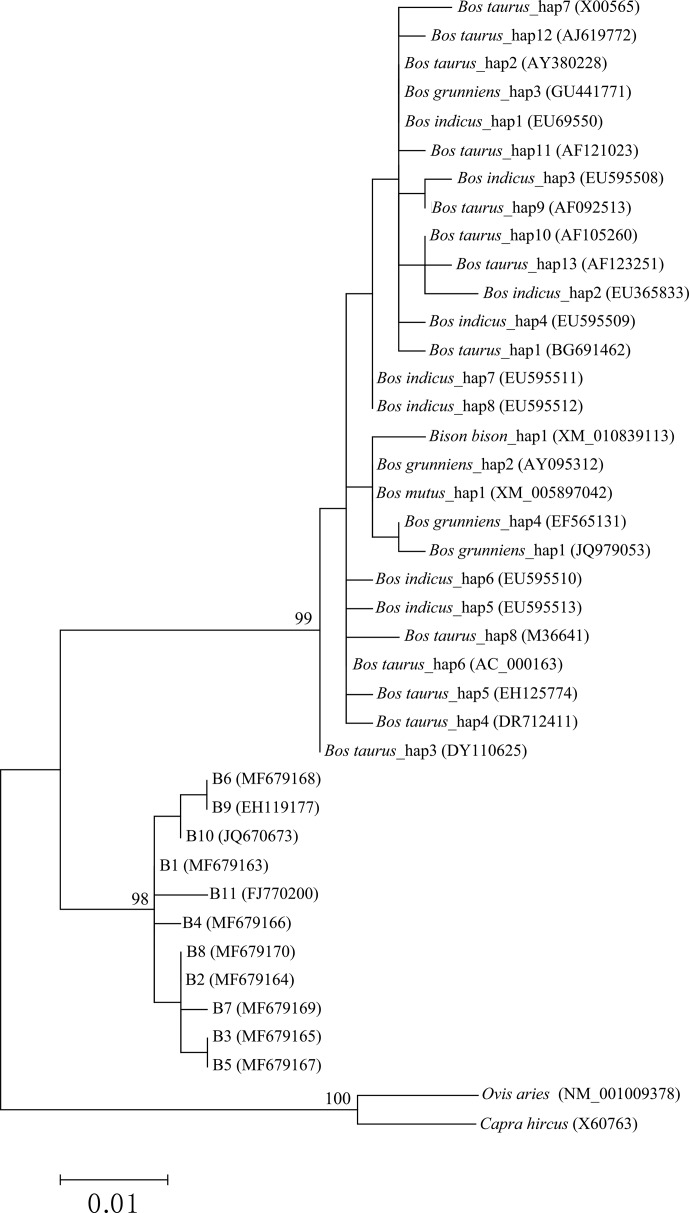
Phylogenetic trees constructed by using the maximum-likelihood method (Hasegawa–Kishino–Yano model). Bootstrap values are based on 10 000 replicates and are adjacent to nodes.

The phylogenetic tree based on the haplotype sequences of the species in the family Bovidae was constructed using the maximum-likelihood method (Fig. 5). The phylogenetic tree was clustered into three principal clades with high support rates. The first clade was formed by *Bos taurus*, *Bos indicus*, *Bos grunniens*, *Bos mutus* and *Bison bison*, indicating a close genetic relationship between these species. The second clade was consisted of two types of buffalo. The third included only goat and sheep.

### 3.5 Inference and nomenclature of buffalo κ-CN variants

Of the eight SNPs identified in buffalo *CSN3*, five (c.86C>T, c.252G>C, c.445G>A, c.467C>T and c.516A>C) were non-synonymous substitutions which caused p.Pro8Leu, p.Lys63Asn, p.Val128Ile, p.Thr135Ile and p.Glu151Asp amino acid substitutions in the mature peptide. Based on these substitutions, we defined seven κ-CN protein variants and four synonymous variants in buffalo. The corresponding relationship between *CSN3* haplotypes and κ-CN variants in buffalo is shown in Table 5. All the κ-CN variants previously identified in the *Bos* genus have not been found in two types of buffalo.

**Table 5:** Amino acid positions and differences in genetic variants of buffalo κ-CN.

κ-CN variant (haplotype)	Position and amino acid in the protein${}^{*}$							
	8	63	66	128	135	136	151	167
Buffalo A (B1)	Pro CCA	Lys AAG	Ala GCA	Val GTT	Thr ACC	Thr ACC	Glu GAA	Thr ACC
Buffalo B (B2)							Asp GAC	
Buffalo B${}^{1}$ (B3)						ACG	Asp GAC	
Buffalo C (B10)					Ile ATC			
Buffalo C${}^{1}$ (B6)					Ile ATC	ACT		
Buffalo C${}^{2}$ (B9)					Ile ATC	ACT		ACG
Buffalo D (B4)				Ile ATT				
Buffalo E (B7)				Ile ATT			Asp GAC	
Buffalo F (B8)	Leu CTA						Asp GAC	
Buffalo F${}^{1}$ (B5)	Leu CTA					ACG	Asp GAC	
Buffalo G (B11)		Asn AAC	GCC					

${}^{*}$ Number represents the position of the mature peptide.

**Figure 6:**
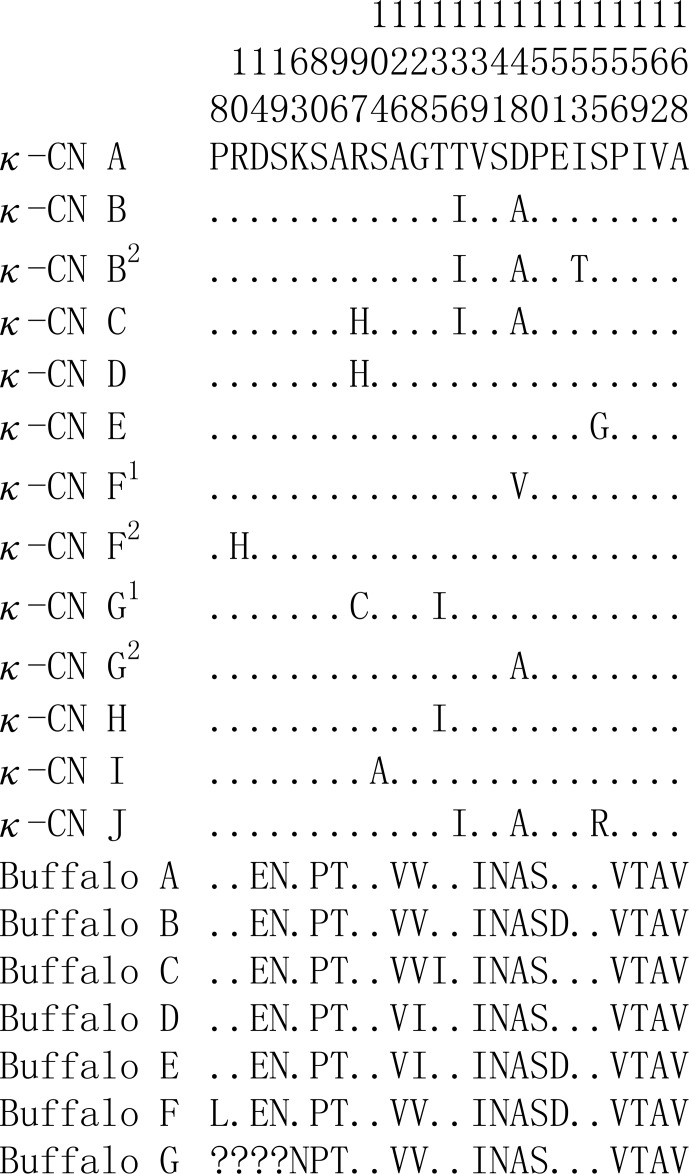
Sequence differences of κ-CN variants between buffalo and the species of the *Bos* genus. Number represents the position of the mature peptide. Dots (⋅) represents the identity with the κ-CN A. Amino acid substitutions are denoted by different letters. Missing information is indicated by a question mark (?).

Based on the literature (Caroli et al., 2009; Gallinat et al., 2013), we have reconstructed the amino acid sequences of the various κ-CN variants found in the *Bos* genus. The sequence alignment of κ-CN variants demonstrated that there were 8 amino acid differential sites within the species of the *Bos* genus, and 14 amino acid differential sites between buffalo and the *Bos* genus (Fig. 6). Because the sequences of κ-CN variants are very different between buffalo and the species of the *Bos* genus, it is not appropriate to include the naming of the buffalo κ-CN variants found in this study in the nomenclature of κ-CN variants of the *Bos* genus. We believe the buffalo variants need to be named independently. Before verifying their existence at the protein level, we suggest the elementary names of buffalo κ-CN variants here, which follow the existing naming conventions for the species of the *Bos* genus in order to improve their rationality. The variants identified in buffalo κ-CN were designated as variants A, B, B${}^{1}$, C, C${}^{1}$, C${}^{2}$, D, E, F, F${}^{1}$ and G, of which variant B${}^{1}$, C${}^{1}$, C${}^{2}$ and F${}^{1}$ are synonymous (Table 5). In this study, the variants A, B, B${}^{1}$, C${}^{1}$, D and F${}^{1}$ were found in river buffalo with frequencies of 84.5 %, 2.6 %, 0.8 %, 7.8 %, 3.4 % and 0.8 %, respectively. The variants A, B, B${}^{1}$, D, E, F and F${}^{1}$ were found in swamp buffalo with frequencies of 40.0 %, 52.1 %, 0.7 %, 4.3 %, 0.7 %, 1.4 % and 0.7 %, respectively.

### 3.6 Functional effect of non-synonymous substitution

The functional effect of non-synonymous substitutions found in the buffalo *CSN3* gene was presumed by the program PROVEAN (Table 6). Only the amino acid substitution p.Thr135Ile was predicted to have an effect on the function of buffalo κ-CN. This substitution changes the theoretical molecular weight of buffalo κ-CN from 19 102.6 to 19 114.7 Da and increases the hydrophobicity, with GRAVY changing from -0.526 (135Thr) to -0.495 (135Ile), and results in the loss of an O-glycosylation site in the corresponding variant of buffalo κ-CN at 135Thr.

**Table 6:** Effect of non-synonymous substitutions on the function of buffalo κ-CN.

SNP	Substitution	PROVEAN	Prediction
		score	(cutoff = -2.5)
c.86C>T	p.Pro8Leu	-2.210	Neutral
c.252G>C	p.Lys63Asn	-2.347	Neutral
c.445G>A	p.Val128Ile	-0.653	Neutral
c.467C>T	p.Thr135Ile	-2.941	Deleterious
c.516A>C	p.Glu151Asp	-2.347	Neutral

## 4 Discussion

κ-CN is an important protein in buffalo milk, accounting for about 12 % of the total casein in buffalo milk (Ahmad et al., 2013). It plays an important role in the formation, size and stability of casein micelles in buffalo milk and further affects the digestibility of milk protein in young animals and the milk processing characteristics of buffalo milk. Previous studies in dairy cows have shown that the polymorphisms of the *CSN3* gene are closely related to milk yield, milk protein and fat content, cheese making, and the curdling characteristics of rennet (Boland et al., 2001; Martin et al., 2002). In this study, the polymorphisms of the *CSN3* gene in river buffalo and swamp buffalo were detected and analyzed in combination with published data of this gene. Eight SNPs were found in water buffalo, including one at exon III and seven at exon IV. Recent studies have shown that the polymorphisms of this gene in other mammals also exist mainly in exon IV (Mukesh et al., 2006). The exon IV of this gene encodes 94.7 % of the mature κ-CN in two types of buffalo and other mammals and constitutes the main part of the κ-casein domain. Therefore, in recent years, the detection of the *CSN3* gene variation in some species mainly focused on exon IV and named κ-CN variants according to the polymorphism of this exon (Farrell et al., 2004; Caroli et al., 2009). In light of this, to study the association between polymorphisms of the *CSN3* gene CDS and the traits in buffalo, it is suggested that exon IV should be used as a genetic marker.

In this study, five amino acid substitutions were observed in the buffalo *CSN3* gene, four of which belonged to amino acid substitutions with the same physical and chemical properties. The predicted results showed that they had no effect on the function of buffalo κ-CN. However, the p.Thr135Ile only found in river buffalo belonged to the substitution of amino acids with different physical and chemical properties, and the evaluation by the program PROVEAN showed that it has an effect on the function of buffalo κ-CN. 135Thr is a probable O-glycosylation site of buffalo κ-CN. This substitution may cause the loss of an O-glycosylation site of buffalo κ-CN. Therefore, we speculate that the substitution may have an important impact on the function of buffalo κ-CN, including the size of casein micelles and coagulation characteristics. Whether this is true or not remains to be further elucidated.

Variants A and B of κ-CN are the most common variants in the animals of the *Bos* genus. Previous studies have focused on genotyping these two variants of the *Bos* genus because they exhibit different biological activities and are associated with milk production traits and cheese making properties (Lara et al., 2002; Tsiaras et al., 2005; Alipanah et al., 2008). Recently, studies in buffalo have used established methods in cattle for *CSN3* genotyping. These studies have endeavored to identify A and B variants in water buffalo with the results demonstrating that the buffalo *CSN3* gene was monomorphic and only had the homozygous allele B. In this study, we did not detect all variants found in cattle, including the two common variants A and B. The buffalo κ-CN seems to contain a recombinant type of “A” and “B” variants of cattle, which was similar to the previous studies in water buffalo (Mitra et al., 1998; Mukesh et al., 2006). In fact, except for SNP467, the other SNPs observed in water buffalo did not exist in the *Bos* genus, and the SNPs observed in the *Bos* genus also did not exist in water buffalo, indicating that the mutation pattern of the *CSN3* gene in water buffalo was obviously different from that in the *Bos* genus. Therefore, the polymorphisms of the *CSN3* gene found in the *Bos* genus cannot simply be applied to water buffalo. The comparison of the haplotypes of the *CSN3* gene showed that there are 15 nucleotide differences between buffalo and the species of the *Bos* genus and 14 amino acid differences at an amino acid level, which indicates that there exist large genetic divergences between them. Phylogenetic analysis also supports that the buffalo *CSN3* is different from that of the species in Bovidae. It is noteworthy that the chymosin hydrolytic site of κ-CN in buffalo and the *Bos* genus is the same, with both located between the 105Phe and 106Met of mature peptide, which reveals the conservation of the chymosin hydrolytic site.

In recent years, the application of molecular biology techniques has enhanced the study of milk protein variants, with a large number of milk protein variants being identified. So far, the κ-CN variants found in the *Bos* genus have been named (Caroli et al., 2009). However, due to the insufficient study of *CSN3* gene polymorphisms in water buffalo, the nomenclature of κ-CN variants in water buffalo has not been fully carried out. In this study, we studied the variation in the *CSN3* gene in water buffalo, trying to reach a comprehensive understanding of its variants. In view of the great difference in the sequences of the *CSN3* gene between buffalo and the *Bos* genus, it is necessary to name the κ-CN variants of buffalo independently. According to the existing nomenclature convention, we named seven κ-CN protein variants and four synonymous variants in water buffalo based on the *CSN3* haplotypes. From the median-joining network of the buffalo haplotypes, it can be seen that buffalo A is probably the ancestral variant of buffalo κ-CN. Buffalo variants C, C${}^{1}$, C${}^{2}$, D and G only have one amino acid difference from variant A, which may be directly derived from buffalo variant A. Buffalo variants C, C${}^{1\;}$and C${}^{2}$ evolved from variant A through amino acid exchange p.Thr135Ile with a synonymous substitution for variants C${}^{1}$ and two synonymous substitutions for C${}^{2}$. Variant D evolved from variant A by amino acid exchange p.Val128Ile and variant G by exchange p.Lys63Asn, while buffalo κ-CN variants B${}^{1}$, E, F and F${}^{1}$ each differ from variant B with one amino acid change and they directly derived from buffalo variant B. Buffalo variant B${}^{1}$ evolved from variant B by a synonymous substitution, and variant F and F${}^{1}$ evolved from variant B by amino acid exchange p.Pro8Leu with an extra synonymous substitution for F${}^{1}$. Variant E evolved from variant B by amino acid exchange p.Val128Ile. As for variants A and B, variant B may be derived from variant A through amino acid exchange p.Glu151Asp. In all the buffalo κ-CN variants identified in this study, since variants E and F were inferred from the lower-frequency haplotypes B7 and B8, respectively, the existence of these two variants needs to be further verified.

The post-translational modification of κ-CN mainly includes O glycosylation and phosphorylation, which plays an important role in its biological function (Bijl et al., 2014). Previous studies have shown that the O glycosylation occurs mainly on the threonine and serine of the macro-peptide formed by chymosin hydrolysis of milk κ-CN, which affects the solubility and biological activity of the macro-peptide and is closely related to the casein micelle size (O'Riordan et al., 2014). Casein micelle size has a direct effect on milk processing characteristics. The results of this study showed that the O-glycosylation sites of buffalo κ-CN were mainly located on threonine and serine of the macro-peptide, which was consistent with the results of previous studies in cattle. The phosphorylation of κ-CN plays an important role in the formation and stabilization of the casein micelle. According to the predicted results, there were differences in the number and types of glycosylation sites and phosphorylation sites of κ-CN between water buffalo and cattle. In addition, 14 amino acid differential sites of κ-CN between buffalo and the *Bos* genus were identified in this study; 3 of them were located at glycosylation sites (80S, 96T, 141S) and 4 at phosphorylation sites (19S, 80S, 96T, 141S). Therefore, we infer that there are some differences in post-translational modification of κ-CN between buffalo and the *Bos* genus, which may lead to differences in the physicochemical properties of their κ-CN.

## 5 Conclusions

In this study, eight SNPs were identified in the buffalo *CSN3* gene, five of which were non-synonymous substitution. The substitution of p.Thr135Ile may have a functional effect on buffalo κ-CN. The mutation pattern of the *CSN3* gene in buffalo was obviously different from that in the *Bos* genus. We defined 11 haplotypes of the buffalo *CSN3* gene. Thus, seven protein variants and four synonymous variants of κ-CN in buffalo were named. The types and frequencies of the variants are different in the two types of buffalo. Furthermore, the variants observed in buffalo did not exist in the *Bos* genus. Whether the SNPs found in this study have any effect on the milk yield and milk composition of buffalo needs to be further studied.

## Data Availability

The original data of the paper are available from the corresponding author upon request.
